# Immunization practices in low birth weight infants from rural Haryana, India: Findings from secondary data analysis

**DOI:** 10.7189/jogh.07.020415

**Published:** 2017-12

**Authors:** Ravi Prakash Upadhyay, Ranadip Chowdhury, Sarmila Mazumder, Sunita Taneja, Bireshwar Sinha, Jose Martines, Rajiv Bahl, Nita Bhandari, Maharaj Kishan Bhan

**Affiliations:** 1 Centre for Health Research and Development, Society for Applied Studies, New Delhi, India; 2 Centre for Intervention Science in Maternal and Child Health, Centre for International Health, University of Bergen, Bergen, Norway; 3 Department of Maternal, Newborn, Child and Adolescent Health, World Health Organization, Geneva, Switzerland; 4 Indian Institute of Technology, New Delhi, India; 5 Knowledge Integration and Translational Platform (KnIT), Biotechnology Industry Research Assistance Council (BIRAC), New Delhi, India

## Abstract

**Background:**

Low birth weight (LBW) infants constitute a vulnerable subset of infants with impaired immunity in early life. In India, there is scarcity of studies that focus on immunization practices in such infants. This analysis aimed to examine immunization practices in LBW infants with the intention to identify areas requiring intervention.

**Methods:**

Data on immunization status of LBW infants enrolled in an individually randomized, double–masked, placebo–controlled trial of neonatal vitamin A supplementation were analysed. Study outcomes were full immunization by one year of age and delayed vaccination with DPT1 and DPT3. Multivariable logistic regression was performed to identify factors associated with the outcome(s).

**Findings:**

Out of 10 644 LBW infants enrolled in trial, immunization data were available for 10 517 (98.8%). Less than one–third (29.7%) were fully immunized by one year of age. Lowest wealth quintile (adjusted odds ratio (AOR) 0.39, 95% confidence interval (CI) 0.32–0.47), Muslim religion (AOR 0.41, 95% CI 0.35–0.48) and age of mother <20 years (AOR 0.62, 95% CI 0.52–0.73) were associated with decreased odds of full immunization. Proportion of infants with delayed vaccination for DPT1 and DPT3 were 52% and 81% respectively. Lowest wealth quintiles (AOR 1.51, 95% CI 1.25–1.82), Muslim religion (AOR 1.41, 95% CI 1.21–1.65), mother aged <20 years (AOR 1.31, 95% CI 1.11–1.53) and birth weight <2000 g (AOR 1.20, 95% CI 1.03–1.40) were associated with higher odds of delayed vaccination for DPT–1. Maternal education (≥12 years of schooling) was associated with high odds of full immunization (AOR 2.39, 95% CI 1.97–2.91) and low odds of delayed vaccination for both DPT–1 (AOR 0.59, 95% CI 0.49–0.73) and DPT–3 (AOR 0.57, 95% CI 0.43–0.76)

**Conclusion:**

In this population, LBW infants are at a risk of delayed and incomplete immunization and therefore need attention. The risks are even higher in identified subgroups that should specifically be targeted

Approximately 15% of infants born in low and middle income countries (LMIC) have a low birth weight (<2500 g) [1]. In India, around 19% of the babies born have a low birth weight [2]. These infants are at a greater risk of morbidity from vaccine–preventable diseases (VPDs) compared to normal birth weight infants (≥2500 g) [3–5]. Immunization is one of the most important and cost–effective public health interventions to reduce both morbidity and mortality associated with infectious diseases [6]. In order to achieve maximal protection, a child should receive all recommended immunizations within specified intervals.

Low birth weight (LBW) infants have a lower passive immunity prior to vaccination and also their immune defences are functionally impaired in early life [5,7,8]. Further, immune protection attained through transplacental transfer of maternal immunoglobulins declines rapidly in these babies, exposing them to an increased risk of infections [4,9,10]. Vaccination has been shown to have a similar efficacy and safety in LBW infants compared to normal birth weight babies. This makes a strong case for these infants to be immunized fully and in time. [4,11].

Previous studies, largely from high income countries, suggest that LBW infants are less likely to receive vaccines on time and be fully immunized [12–14]. The proposed reasons were high rates of medical complications, leading to prolonged hospitalization; lack of awareness among parents about benefits of vaccination and concerns about possible harm to these infants, perceived to be feeble and delicate [15–18]. In LMICs, studies have mostly examined immunization coverage and their determinants in children above 12 months, irrespective of their birth weight but such studies do not widen our horizon of understanding of immunization practices in LBW infants, that form a vulnerable subset [19,20].

Recently, few studies have been conducted that document immunization practices in LBW infants from rural Ghana [21,22]. However, in India, where high burden of such babies is of concern, lack of systematic studies obscures our understanding of their immunization practices. This information is essential in order to inform public health policy so that special efforts could be undertaken to improve uptake of immunization services in low birth weight infants. With this background, current secondary analysis was planned to document the immunization practices and their determinants in LBW infants, using data from an individually randomized, double–masked, placebo–controlled trial in rural Haryana, India [23,24]. As a secondary objective, we examined the association of birth weight with immunization practices.

## MATERIALS AND METHODS

### Study design and setting

The present analysis utilizes data on the immunization status of low birth weight infants enrolled in a large individually randomized, double–masked, placebo–controlled trial of neonatal vitamin A supplementation within 72 hours of birth. This study was conducted in Faridabad and Palwal districts in the state of Haryana, North India from June 2010 to July 2012 [23]. The trial procedures and details of study area have been described previously [23,24].

### Ethical clearance

The trial was approved by the ethics review committees of the Society for Applied Studies, World Health Organization (WHO) and by the state government of Haryana. It is registered with ClinicalTrials.gov, number NCT01138449. Permission was taken from all the concerned investigators of the primary trial for this secondary data analysis

### Enrolment and data collection

Study teams identified pregnant women through household surveillance at intervals of 3 months in areas allocated to them. The pregnancies identified were followed up until delivery and birth outcomes were reported to the co–ordinators who then informed the enrolment workers immediately [24]. For each live birth identified, the study team visited the family, explained the trial, screened the infant against a pre–defined eligibility criteria (infants aged ≤3 days at screening, could suck or feed and family intended to stay in the study area for at least 6 months) and obtained written consent from at least one parent ie, mother/ father of eligible infants. The enrolled infant was weighed by the study team members who were trained and standardized.

At enrolment, information was collected primarily on household characteristics (social class, religion, wealth quintile), infant characteristics (birth weight, sex, place of delivery, personnel conducting delivery, multiple births), maternal characteristics (number of living children, age, education, occupation) and father’s education. Each enrolled infant was allocated a home visit worker for further follow up until 12 months of age. All infants were contacted when aged 29 days and at 3, 6 and 12 months and at each such visit, information was collected on vital status and immunization.

At each visit, the study team member looked for written documentation of vaccines administered to the infant. The documents reviewed were maternal and child health card, immunization card of the infant or any slip(s) issued by the facility where vaccination was done. The study team made several attempts to obtain written documented evidence of vaccination. This included a wait time, to ensure the mother ample time to search for the missing record, telephoning the father for any relevant information, and also postponing the visit to a later date. If the immunization card was still not available the team helped mother to report accurate dates by referring to important events or festivals. Also, the mother was asked to recall which vaccines were given, at what body site and the mode of vaccination (oral or injection). An infant was categorised as “not vaccinated” when the mother reported infant had never been vaccinated.

### Outcomes of the secondary analysis

The primary outcomes were full immunization by one year of age and delayed vaccination with DPT1 and DPT3 in LBW infants. In concordance with the guidelines of the National Immunization Program in India, an infant was considered “fully immunized” if he/she had received BCG, 3 doses of DPT, OPV each and measles by one year of age [25]. Hepatitis–B immunization was not considered as a part of full immunization as the vaccine was not introduced during the time of trial in the state of Haryana [26]. There is no standard approach to the assessment of delayed vaccination and several definitions have been described [27,28]. However, previous studies have considered DPT–1 and DPT–3 vaccination as acceptable points to assess delay [13,21].

Operationally, “delayed vaccination” was defined as having received the vaccine after 4 weeks of recommended/due time [21,29,30]. In India BCG is to be given at birth; OPV–1 and DPT–1 at 6 weeks of age; OPV–2 and DPT–2 at 10 weeks of age, OPV–3 and DPT–3 at 14 weeks of age; measles at 9 months of age [25]. For the primary analysis, a delay in DPT–1 was considered when the LBW infant was vaccinated later than 10 weeks of age and for DPT–3, when the infant was vaccinated at >18 weeks of age.

Additionally, sensitivity analysis was done to assess whether delayed DPT3 vaccination reflected delayed DPT1 vaccination. Starting with follow–up at receipt of DPT1 vaccination, an infant was labelled as having a “delayed receipt” of DPT–3 when it was given >12 weeks after DPT–1 (according to National Immunization Schedule, the time interval between DPT–1 and DPT–3 should be 8 weeks).

### Data analysis

For the analyses, infants with known vaccination status, dates of vaccination and with complete data on covariates were included. Infants who were lost to follow–up or died before the vaccination due date, were excluded. This principle was followed for all the time points of analysis. Data analysis was performed using STATA version 11 (Stata Corporation, College Station, TX). Proportions were calculated for all categorical variables used in the analysis. Median (interquartile range; IQR) was calculated for delay in vaccination (in days), from the recommended time, for each of the vaccine that was considered in the analysis. Chi–square test was done to compare proportions and Wilcoxon–Mann–Whitney 2– sample rank sum test to compare medians across the two birth weight categories.

Multivariable logistic regression was performed to identify factors associated with full immunization and delayed vaccination. Bivariate analysis was first done for all explanatory variables and those with a *P*–value of <0.20 were then included in the final multivariable logistic regression model [31,32]. A *P*–value of <0.05 was considered statistically significant in the final regression model. Explanatory variables considered were household characteristics (wealth index, religion. social class); maternal and paternal characteristics (maternal age, maternal education, maternal occupation, paternal education); birth related characteristics (place of delivery, personnel conducting delivery, multiple births, and number of living children) and infant characteristics (birth weight and sex).

Additionally, to assess the association of birth weight on study outcome(s) ie, “full immunization “and “delayed vaccination”, regression analysis was done with birth weight as the exposure variable (in dichotomous form ie, ≥2500 and <2500 g) and adjustment done for other covariates. Assessment for effect modification (ie, potential interaction) between birth weight and all covariates was done using interaction term in the model. Likelihood ratio test was used to compare models with or without the interaction term. Sensitivity analysis was also conducted where data collected only from immunization cards were analysed to document the determinants of full immunization and delayed vaccination in low birth weight infants. Analysis to document the determinants of full immunization and delayed vaccination in normal birth weight infants was also undertaken on an exploratory basis.

## RESULTS

### Characteristics of the infants recruited in the primary trial

A total of 44 984 infants were recruited in the primary trial, within 72 hours of birth, out of which 10 644 (23.7%) were low birth weight infants. This subset of LBW infants was analysed for the primary outcome(s). However, to give a general sense of how these low birth weight infants compared to their normal birth weight counterparts, a comparative description of the characteristics have been presented (**Table 1**). The proportion of female infants was more in the LBW category (55.5%) compared to normal birth weight (NBW) infants (45.6%) (**Table 1**). The mean (SD) birth weight of normal and low birth weight infants was 2914.0 (421.0) and 2193.1 (224.3) grams respectively. A similar proportion of LBW and NBW infants were born at home (44.6% vs 42.8%). Most of the LBW infants had mothers with low literacy (50.3% of mothers reported having no education or not completing primary school) and 11% had mothers with age less than 20 years (**Table 1**). In NBW infants, low maternal literacy was observed in 44.4% and around 7% (2388/34,340) had mothers aged less than 20 years.

**Table 1:** Baseline characteristics of the primary trial population, segregated by low and normal birth weight infants (N = 44 984)

Variables	Normal birth weight (≥2500 g; n = 34 340)		Low birth weight (<2500 g; n = 10 644)
**Household characteristics**			
**Quintiles**:*			
1 (Least poor)	7391 (21.5)		1613 (15.1)
2	7043 (20.5)		1947 (18.3)
3	6873 (20.0)		2124 (20.0)
4	6628 (19.3)		2369 (22.3)
5 (Poorest)	6405 (18.7)		2591 (24.3)
**Religion**:*			
Hindu	26 401 (76.9)		8171 (76.8)
Muslim	7582 (22.1)		2323 (21.8)
Others	357 (1.0)		148 (1.4)
**Social class**:*†			
General	9587 (27.9)		2453 (23.1)
Other Backward Class (OBC)	16 583 (48.3)		5308 (49.8)
Scheduled Caste/Tribe (SC/ST)	8170 (23.8)		2881 (27.1)
**Maternal and paternal characteristics**			
**Mother’s age** (in years):*			
<20	2388 (6.9)	1175 (11.0)	
20–25	22 705 (66.1)	7097 (66.7)	
26–30	7159 (20.9)	1784 (16.8)	
>30	2088 (6.1)	588 (5.5)	
**Mother’s education** (years of schooling):*			
Illiterate (0)	13 895 (40.5)	4918 (46.2)	
Less than primary (1 to <5)	1351 (3.9)	433 (4.1)	
Primary completed and secondary incomplete (5 to <12)	14 847 (43.2)	4418 (41.5)	
Secondary complete and higher education (≥12)	4247 (12.4)	875 (8.2)	
**Mother’s occupation:**			
Employed outside home	888 (2.6)	252 (2.4)	
Home maker	33 452 (97.4)	10 392 (97.6)	
**Father’s education** (years of schooling):*			
Illiterate (0)	4367 (12.7)	1652 (15.5)	
Less than primary (1 to <5)	1617 (4.7)	600 (5.7)	
Primary completed and secondary incomplete (5 to <12)	19 396 (56.5)	6336 (59.5)	
Secondary complete and higher education (≥12)	8960 (26.1)	2056 (19.3)	
**Birth related characteristics**			
**Place of delivery**:*			
Home	14 694 (42.8)	4753(44.6)	
Government facility	10 863 (31.6)	3273 (30.8)	
Private facility	8783 (25.6)	2618 (24.6)	
**Personnel conducting delivery**:*			
Skilled	21 187 (61.7)	6371 (59.9)	
Unskilled	13 153 (38.3)	4273 (40.1)	
**No. newborns**:*			
Singleton	34 245 (99.7)	10 168 (95.5)	
Multiple	95 (0.3)	476 (4.5)	
**No. of living children** (apart from the infant enrolled):*‡			
0	10 501 (30.6)	4226 (39.7)	
1–2	17 489 (50.9)	4938 (46.4)	
3–4	3293 (9.6)	761 (7.1)	
≥4	3057 (8.9)	719 (6.8)	
**Infant characteristics**			
**Sex of the baby**:*			
Male	18 676 (54.4)		4742 (44.5)
Female	15 664 (45.6)		5902 (55.5)

*Statistically significant difference in proportion across the two groups (*P* < 0.05); Others – Christian/Sikh/Jain/Parsi/Zoroastrian/Buddhist/neo Buddhist.

†General – group that do not qualify for any of the positive discrimination schemes by Government of India (GOI); OBC – term used by the Government of India to classify castes which are socially and educationally disadvantaged; SC/ST – official designations given to groups of historically disadvantaged indigenous people in India [33].

‡Excluding the baby recently born/enrolled in the study.

**Figure 1:**
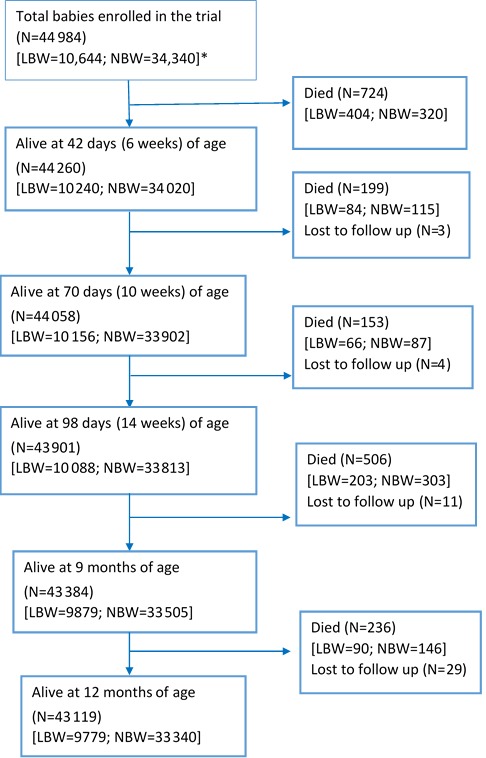
Flow of infants recruited in the primary trial. *LBW – low birth weight; NBW – normal birth weight. The flow shows the number of alive babies at 6, 10, 14 weeks and at 9 and 12 months of age specifically with the intention of present the number of babies eligible for OPV1/DPT1 (given at 6 weeks), OPV2/DPT2 (given at 10 weeks), OPV3/DPT3 (given at 14 weeks) and Measles (given at 9 months of age).

**Figure 1** shows the flow of infants recruited in the primary trial, starting from the time of recruitment ie, within 2 days of birth, until the age of 1 year. Out of the 10 644 LBW infants that were enrolled, 847 died by the end of one year (infant mortality rate of 79.6/1000 live births). A large proportion of the deaths occurred in the first 6 weeks of life (n = 404/847; 47.4%). In normal birth weight infants, there were a total of 971 deaths in the first year of life, resulting in an infant mortality rate of 28.3/1000 live births.

### Immunization practices among the low birth weight infants

Out of the total 10 644 LBW infants that were enrolled in the trial, immunization data was available for 10 517 (98.8%). In 77.8% infants, data was obtained through “immunization card” and in rest; it was elicited through reliable history. Low birth weight infants had a comparatively lower immunization uptake compared to normal weight infants, both in terms of the proportion that received a particular vaccine and also in appropriateness of timing of receiving vaccine (**Table 2**). Around three–fourth received BCG vaccine (75.9%) and one–fifth (20.3%) received zero–dose of polio vaccine. The proportion that received DPT–1, DPT–2, DPT–3 and measles vaccine was 74.1%, 58.3%, 45.4% and 35.6% respectively. Less than one–third (29.7%) were fully immunized by one year of age ie, had received BCG, three doses of DPT and OPV each and measles vaccine.

**Table 2:** Immunization uptake among normal birth weight (≥2500 g) and low birth weight (<2500 g) babies in rural Haryana, North India

Vaccines under National Immunization Schedule	Number*			Proportion received (%)			Deviation from recommended time (days) of vaccination, median (IQR)**†**			Proportion with delay (%)		
**LBW**	**NBW**	**Overall**	**LBW**	**NBW**	**Overall**	**LBW**	**NBW**	**Overall**	**LBW**	**NBW**	**Overall**
BCG	10 517	34 262	44 779	75.9‡	80.8	79.6	41 (19–75)‡	39 (18–70)	39 (18–71)	64.4‡	62.7	63.1
OPV–0§				20.3‡	22.2	21.8	–	–	–	–	–	–
OPV–1	10 240	34 020	44 260	64.4‡	68.9	67.9	27 (10–56)‡	24 (9–52)	25 (10–53)	48.1‡	44.6	45.4
DPT–1				74.1‡	78.3	77.3	30 (12–63)‡	27 (11–58)	27 (11–59)	51.7‡	47.9	48.7
OPV–2	10 156	33 902	44 058	51.5‡	57.5	56.1	44 (21–84)‡	41 (20–77)	42 (20–79)	65.7‡	63.6	64.0
DPT–2				58.3‡	63.9	62.7	46 (23–89)‡	44 (21–82)	44 (21–84)	67.8‡	65.5	65.9
OPV–3	10 088	33 813	43 901	40.8‡	46.6	45.3	60 (33–107)‡	59 (31–101)	59 (32–102)	79.6‡	77.8	78.2
DPT–3				45.4‡	51.2	49.9	62 (34–112)‡	60 (32–104)	61 (32–105)	80.7‡	78.7	79.1
Measles (at 9 months)	9879	33 505	43 384	35.6‡	41.2	39.9	24 (9–46)‡	22 (8–43)	22 (8–44)	43.4‡	41.1	41.6
Fully immunized at 1 year of age	9779	33 340	43 119	29.7‡	35.2	34.0	–	–		–	–	

LBW – low birth weight, NBW – normal birth weight

*For 205 babies (all died in the first month of life) information on immunization was not available, 724 babies died at or before 42 days (6 weeks) of age, 923 died at or before 70 days (10 weeks) of age, 1076 died at or before 98 days (14 weeks) of age, 1582 died at or before 270 days (9 months) of age, 1818 died within one year.

†According to the National Immunization Schedule (Government of India). BCG and OPV-0 at birth, OPV-1/DPT-1 at 6 weeks of age, OPV-2/DPT-2 at 10 weeks of age, OPV-3/DPT-3 at 14 weeks of age and Measles at 9 months of age.

‡Statistically significant difference (*P*<0.05) compared to normal birth weight babies.

§Data not available on the time of receiving of birth dose of polio vaccine.

There was a delay in the time of receipt of the vaccines compared to the recommended time as per the National Immunization Schedule. The median (interquartile range; IQR) delay for BCG, DPT–1, DPT–2 and DPT–3 was 41 (19–75), 30 (12–63), 46 (23–89) and 62 (34–112) days respectively. Around 65% of the LBW babies had delay in receiving BCG, 52% in DPT–1, 68% in DPT–2 and 81% in DPT–3. For measles vaccine, the median (IQR) delay from the recommended time was 24 (9–46) days and around two–fifth infants (43.5%) had delay in receiving the vaccine.

### Determinants of full immunization in LBW babies

Out of 9779 LBW infants that were alive at the age of 1 year, 2913 (29.7%) were fully immunized. There was a dose response relationship between wealth quintiles and full immunization status. Those LBW infants who were from the lowest wealth quintile had the lowest odds compared to those in highest wealth quintile (adjusted odds ratio (AOR) 0.39; 95% confidence interval (CI), 0.32–0.47] (**Table 3**). Belonging to a Muslim family (AOR 0.41; 95% CI, 0.35–0.48), mother’s age <20 years (AOR 0.62; 95% CI, 0.52–0.73) delivered by unskilled attendant (AOR 0.77; 95% CI, 0.64–0.91) and being a female (AOR 0.84; 95% CI, 0.77–0.92) decreased the odds. With increasing number of living children a women had, the odds of fully immunizing the recently delivered LBW baby decreased; the lowest odds in those with ≥4 children (AOR 0.58; 95% CI, 0.43–0.77) (**Table 3**).

**Table 3:** Determinants of full immunization at one year of age among low birth weight babies in rural Haryana, North India

Variables	Unadjusted OR (95% CI)	*P*–value	Adjusted OR (95% CI)*	*P* –value
**Household characteristics**				
**Quintiles:**				
1 (Least poor)	Ref.		Ref.	
2	0.70 (0.61–0.81)	<0.001	0.83 (0.71–0.95)	0.010
3	0.45 (0.38–0.51)	<0.001	0.61 (0.52–0.71)	<0.001
4	0.35 (0.30–0.40)	<0.001	0.54 (0.46–0.64)	<0.001
5 (Poorest)	0.19 (0.16–0.22)	<0.001	0.39 (0.32–0.47)	<0.001
**Religion:**				
Hindu	Ref.		Ref.	
Muslim	0.24 (0.21–0.28)	<0.001	0.41 (0.35–0.48)	<0.001
Others†	0.96 (0.67–1.37)	0.824	1.23 (0.84–1.79)	0.284
**Social class:**				
General	Ref.		Ref.	
Other Backward Class	0.52 (0.46–0.57)	<0.001	1.10 (0.97–1.24)	0.122
Scheduled Caste/Tribe	0.75 (0.67–0.84)	<0.001	1.30 (1.14–1.49)	<0.001
**Maternal and paternal characteristics**				
**Mother’s age (in years):**				
<20	0.59 (0.51–0.69)	<0.001	0.62 (0.52–0.73)	<0.001
20–25	Ref.		Ref.	
26–30	0.94 (0.83–1.06)	0.305	1.38 (1.19–1.58)	<0.001
>30	0.62 (0.49–0.77)	<0.001	1.49 (1.15–1.95)	0.003
**Mother’s education (years of schooling):**				
Illiterate (0)	Ref.		Ref.	
Less than primary (1 to <5)	1.48 (1.16–1.88)	0.001	1.23 (0.96–1.58)	0.105
Primary completed and secondary incomplete (5 to <12)	2.61 (2.36–2.89)	<0.001	1.56 (1.39–1.75)	<0.001
Secondary complete and higher education (≥12)	5.56 (4.76–6.50)	<0.001	2.39 (1.97–2.91)	<0.001
**Father’s education (years of schooling):**				
Illiterate (0)	Ref.		Ref.	0.203
Less than primary (1 to <5)	1.27 (0.97–1.66)	0.075	1.19 (0.91–1.58)	<0.001
Primary completed and secondary incomplete (5 to <12)	2.53 (2.17–2.95)	<0.001	1.53 (1.29–1.81)	<0.001
Secondary complete and higher education (≥12)	4.51 (3.80–5.35)	<0.001	1.49 (1.22–1.83)	
**Birth related characteristics**				
**Place of delivery**:				
Home	Ref.		Ref.	
Government facility	1.97 (1.78–2.19)	<0.001	1.29 (1.08–1.54)	0.004
Private facility	1.98 (1.77–2.22)	<0.001	0.96 (0.79–1.15)	0.649
**Personnel conducting delivery**:‡				
Skilled	Ref.		Ref.	
Unskilled	0.45 (0.41–0.48)	<0.001	0.77 (0.64–0.91)	0.003
**No. of living children:**				
0	Ref.		Ref.	
1–2	0.86 (0.78–0.95)	0.002	0.89 (0.79–0.98)	0.031
3–4	0.56 (0.46–0.68)	<0.001	0.70 (0.56–0.88)	0.002
>4	0.32 (0.26–0.41)	<0.001	0.58 (0.43–0.77)	<0.001
**No. newborns**:				
Singleton	Ref.		Ref.	
Multiple	1.29 (1.04–1.61)	0.019	1.14 (0.89–1.45)	0.302
**Infant characteristics**				
**Birth weight (in grams):**				
2000–2499	Ref.		Ref.	
<2000	0.88 (0.76–1.03)	0.104	0.88 (0.75–1.03)	0.122
**Sex of the baby**:				
Male	Ref.		Ref.	
Female	0.86 (0.78–0.93)	<0.001	0.84 (0.77–0.92)†	<0.001

OR – odds ratio, Ref. – reference value

*Variables with *P*–value <0.20 in the bivariate analysis were included in the multivariable analysis and have been presented in the table. Mother’s occupation had a *P*–value of ≥0.20 in bivariate analysis and was not included in the multivariable analysis

† Others – Christian/Sikh/Jain/Parsi/Zoroastrian/Buddhist/neo Buddhist.

‡Skilled attendant included doctor/nurse/Auxiliary Nurse Midwife/community health worker; unskilled included traditional birth attendant/relative/neighbour.

Compared to infants with illiterate parents, those with mothers [AOR 2.39; 95% CI, 1.97–2.91] and fathers [AOR 1.49; 95% CI 1.22–1.83] who were educated until secondary school or higher (≥12 years of schooling) had increased odds of full immunization. Mother’s age was also an important determinant. Compared to mother’s aged 20–25 years, those aged 26–30 years [AOR 1.38; 95% CI, 1.19–1.58] and >30 years [AOR 1.49; 95% CI, 1.15–1.95] had higher odds of getting their child fully immunized. Also, delivery at a government health facility [AOR 1.29; 95% CI, 1.08–1.54] increased the odds (**Table 3**).

In the sensitivity analysis, using data documented through immunization cards, 15.2% of LBW infants were fully immunized by 12 months of age. The determinants of full immunization in these infants were essentially similar to those obtained when combined data obtained through immunization cards and reliable histories were analysed (Table S1 in **Online** **Supplementary Document**). Lower wealth quintile, belonging to a Muslim family, mother’s age <20 years, delivered by unskilled attendant, ≥4 living children for the woman and female sex of the infant were associated with decreased odds. Unlike in the combined analysis wherein father’s education was associated with increased odds of the child for being full immunized, in the sensitivity analysis, father’s education did not emerge as a statistically significant determinant. However, mother’s education and delivery at the government health facility were associated with increased odds.

### Determinants of delayed vaccination in LBW babies

Lowest wealth quintiles [AOR 1.51; 95% CI, 1.25–1.82], Muslim religion (AOR 1.41; 95% CI, 1.21–1.65), mother aged <20 years (AOR 1.31; 95% CI, 1.11–1.53) and birth weight <2000 g (AOR 1.20; 95% CI, 1.03–1.40) were associated with higher odds of delayed vaccination with first–dose of DPT (DPT–1) vaccine (**Table 4**). On the other hand, higher maternal education (AOR 0.59; 95% CI, 0.49–0.73) and delivery in a government facility (AOR 0.81; 95% CI, 0.68–0.96) were associated with lower odds of delay for DPT–1. Interestingly, maternal education status was the only variable that was significantly associated with delay in receiving third dose of DPT (DPT–3). Infants of mothers with ≥12 years of schooling (ie, secondary school complete and higher education) had lower odds of delay (AOR 0.57; 95% CI, 0.43–0.76) for DPT–3 compared to those with illiterate mothers (**Table 4**).

**Table 4:** Determinants of delayed vaccination with first dose DPT at age >10 weeks and third dose DPT at age >18 weeks for low birth weight babies in rural Haryana, North India

Variables	DPT–1 (at >10 weeks after birth)				DPT–3 (at >18 weeks after birth)			
**Unadjusted OR (95% CI)**	***P*–value**	Adjusted OR (95% CI)*	***P*–value**	**Unadjusted OR (95% CI)**	***P*–value**	Adjusted OR (95% CI)*	***P*–value**
**Household characteristics**								
**Quintiles**:								
1 (Least poor)	Ref.		Ref.		Ref.		Ref.	
2	1.36 (1.18–1.57)	<0.001	1.26 (1.09–1.47)†	0.002	1.19 (0.96–1.47)	0.110	1.04 (0.83–1.30)	0.742
3	1.49 (1.29–1.72)	<0.001	1.32 (1.13–1.55)†	0.001	1.04 (0.83–1.29)	0.736	0.84 (0.65–1.07)	0.150
4	1.48 (1.28–1.71)	<0.001	1.27 (1.08–1.51)†	0.005	1.07 (0.86–1.34)	0.537	0.83 (0.64–1.08)	0.164
5 (Poorest)	1.93 (1.66–2.24)	<0.001	1.51 (1.25–1.82)†	<0.001	1.20 (0.93–1.55)	0.157	0.83 (0.61–1.14)	0.266
**Religion**:								
Hindu	Ref.		Ref.		Ref.		Ref.	
Muslim	1.66 (1.44–1.90)	<0.001	1.41 (1.21–1.65)†	<0.001	1.31 (0.99–1.73)	0.055	1.06 (0.79–1.43)	0.680
Others‡	1.17 (0.81–1.70)	0.404	1.12 (0.77–1.64)	0.554	1.78 (0.88–3.59)	0.106	1.59 (0.79–3.24)	0.194
**Social class**:								
General	Ref.		Ref.		Ref.		Ref.	
Other Backward Class	1.28 (1.15–1.43)	<0.001	1.00 (0.89–1.13)	0.990	1.28 (1.07–1.53)	0.006	1.17 (0.96–1.41)	0.113
Scheduled Caste/Tribe	1.18 (1.04–1.33)	0.008	0.97 (0.85–1.11)	0.704	0.97 (0.81–1.17)	0.783	0.89 (0.72–1.08)	0.243
**Maternal and paternal characteristics**								
**Mother’s age (in years):**								
<20	1.40 (1.21–1.63)	<0.001	1.31 (1.11–1.53)†	0.001	1.36 (1.04–1.81)	0.027	1.31 (0.97–1.73)	0.060
20–25	Ref.		Ref.		Ref.		Ref.	
26–30	1.05 (0.92–1.19)	0.475	0.95 (0.83–1.09)	0.467	0.98 (0.81–1.21)	0.906	0.97 (0.79–1.19)	0.792
>30	1.22 (0.97–1.52)	0.080	0.92 (0.71–1.19)	0.521	1.08 (0.72–1.61)	0.706	0.97 (0.64–1.45)	0.871
**Mother’s education (years of schooling):**								
Illiterate (0)	Ref.		Ref.		Ref.		Ref.	
Less than primary (1 to <5)	0.82 (0.64–1.05)	0.116	0.87 (0.68–1.11)	0.274	0.76 (0.51–1.13)	0.177	0.77 (0.51–1.15)	0.212
Primary completed and secondary incomplete (5 to <12)	0.76 (0.68–0.84)	<0.001	0.86 (0.77–0.97)†	0.014	0.88 (0.74–1.05)	0.162	0.86 (0.71–1.05)	0.131
Secondary complete and higher education (≥12)	0.47 (0.39–0.55)	<0.001	0.59 (0.49–0.73)†	<0.001	0.58 (0.46–0.72)	<0.001	0.57 (0.43–0.76)†	<0.001
**Father’s education** (years of schooling):								
Illiterate (0)	Ref.		Ref.		Ref.		Ref.	
Less than primary (1 to <5)	1.02 (0.79–1.32)	0.857	1.06 (0.82–1.37)	0.648	0.86 (0.54–1.38)	0.547	0.91 (0.56–1.47)	0.703
Primary completed and secondary incomplete (5 to <12)	0.84 (0.73–0.97)	0.018	1.02(0.87–1.19)	0.831	0.87 (0.66–1.15)	0.328	0.93 (0.69–1.25)	0.634
Secondary complete and higher education (≥12)	0.67 (0.67–0.78)	<0.001	1.07 (0.88–1.29)	0.502	0.72 (0.53–0.95)	0.025	0.87(0.62–1.21)	0.406
**Birth related characteristics**								
**Place of delivery**:								
Home	Ref.		Ref.		Ref.		Ref.	
Government facility	0.68 (0.61–0.75)	<0.001	0.81 (0.68–0.96)†	0.017	0.72 (0.60–0.86)	<0.001	0.79 (0.60–1.06)	0.126
Private facility	0.82 (0.73–0.92)	0.001	1.08 (0.91–1.29)	0.377	0.79 (0.66–0.95)	0.014	0.88 (0.66–1.18)	0.409
**Personnel conducting delivery**:§								
Skilled	Ref.		Ref.		Ref.		Ref.	
Unskilled	1.41 (1.28–1.55)	<0.001	1.15 (0.96–1.37)	0.114	1.32 (1.11–1.56)	0.001	1.08 (0.81–1.45)	0.589
**No. of living children**:							–	–
0	Ref.		Ref.		Ref.			
1–2	0.95 (0.86–1.05)	0.312	0.93 (0.84–1.04)	0.204	0.97 (0.84–1.13)	0.725		
3–4	1.28 (1.05–1.56)	0.013	1.14 (0.92–1.43)	0.235	1.24 (0.86–1.77)	0.247		
≥4	1.37 (1.09–1.72)	0.006	1.09 (0.83–1.43)	0.544	1.18 (0.75–1.85)	0.473		
**No. newborns**:			–	–		0.977	–	–
Singleton	Ref				Ref			
Multiple	0.98 (0.78–1.23)	0.878			0.99 (0.69–1.42)			
**Infant characteristics**								
**Birth weight (in grams):**								
2000–2499	Ref.		Ref.		Ref.		Ref.	
<2000	1.23 (1.06–1.43)	0.007	1.20 (1.03–1.40)†	0.019	1.06 (0.83–1.37)	0.602	1.04 (0.81–1.34)	0.774
**Sex of the baby**:								
Male	Ref.		–	–	Ref.		–	–
Female	0.95 (0.87–1.05)	0.340			0.92 (0.79–1.07)	0.275		

*Variables with *P*-value <0.20 in the bivariate analysis were included in the multivariable analysis and have been presented in the table, exception being the birth weight which was placed in the multivariable regression model irrespective of the p-values in bivariate analysis as this was considered an essential variable determining delay. Mother’s occupation had a *P*-value of ≥0.20 in bivariate analysis and was not included in the multivariable analysis.

†Statistically significant at P<0.05.

‡Christian/Sikh/Jain/Parsi/Zoroastrian/Buddhist/neo Buddhist.

§Skilled attendant included doctor/nurse/Auxiliary Nurse Midwife/community health worker; unskilled included traditional birth attendant/relative/neighbour.

In the sensitivity analysis, using data obtained through immunization cards, 46.6% and 79.2% of the LBW infants had delay in vaccination for DPT–1 and DPT–3 respectively. Lower wealth quintiles, Muslim religion, mother aged <20 years and birth weight <2000 g were associated with higher odds of delayed vaccination with first–dose of DPT (DPT–1) vaccine (Table S2 in **Online Supplementary Document(Online Supplementary Document)**). On the other hand, higher maternal education (AOR 0.56; 95% CI, 0.45–0.71) and delivery in a government facility (AOR 0.71; 95% CI, 0.57–0.86) were associated with lower odds of delay for DPT–1. For DPT–3, higher maternal education was associated with reduced odds of delayed vaccination (AOR 0.56; 95% CI, 0.41–0.76).

### Birth weight as a determinant of full immunization and delayed vaccination

Low birth weight was associated with reduced odds of full immunization (AOR 0.85; 95% CI, 0.81–0.90) (**Table 5**). For both DPT–1 and DPT–3, a statistically significant interaction was obtained between birth weight and sex (*P*–value for interaction = 0.0006 and 0.020 respectively). Low birth weight was associated with increased odds of delayed vaccination for DPT–1 (AOR 1.18; 95% CI, 1.10–1.28) and DPT–3 (AOR 1.18; 95% CI, 1.04–1.33) in male infants but there was no such significant association in female infants. After adjusting for the late vaccination with DPT–1, birth weight had no significant association with delay in DPT–3 vaccine (**Table 5**).

**Table 5:** Birth weight as a determinant of full immunization and delayed vaccination in infants from rural Haryana, North India

Variables	Proportion (%)	Unadjusted OR (95% CI)	*P–*value	Adjusted OR (95% CI)*	*P–*value
**Full immunization**	**Fully immunized (n) / Alive at 1 year (No.); (%)**				
Birth weight (grams):					
≥2500	11760/33340; (35.2)	Ref.		Ref.	
<2500	2913/9779; (29.7)	0.78 (0.74–0.82)	<0.001	0.85 (0.81–0.90)	<0.001
**Delayed vaccination**	**No. with delayed vaccination (n) / total No. that received the vaccine (N); (%)**				
**DPT–1 (at >10 weeks) by sex of the infant (*P–*value for interaction = 0.0006)**					
Male infants – Birth weight (grams):					
≥2500	6835/14737; (46.3)	Ref.		Ref.	
<2500	1800/3443; (52.3)	1.27 (1.17–1.36)	<0.001	1.18 (1.10–1.28)	<0.001
Female infants – Birth weight (grams):					
≥2500	5918/11892; (49.7)	Ref.		Ref.	
<2500	2123/4148; (51.2)	1.05 (0.98–1.14)	0.116	1.02 (0.94–1.09)	0.592
**DPT–3 (at >18 weeks) by sex of the infant (***P***–value for interaction = 0.020)**					
Male infants – Birth weight (grams):					
≥2500	7639/9818; (77.8)	Ref.		Ref.	
<2500	1737 /2135; (81.3)	1.24 (1.11–1.40)	<0.001	1.18 (1.04–1.33)	0.008
Female infants – Birth weight (grams):					
≥2500	5986/7513; (79.6)	Ref.		Ref.	
<2500	1962/2450; (80.1)	1.03 (0.92–1.15)	0.664	0.99 (0.88–1.12)	0.984
**DPT–3 after 12 weeks of DPT–1†**					
Birth weight (grams):					
≥2500	9207/17315; (53.1)	Ref.		Ref.	
<2500	2506/4579; (54.7)	1.06 (0.99–1.14)	0.061	1.04 (0.97–1.12)	0.183

*Adjusted for infant sex, multiple births, place of delivery, personnel conducting delivery (skilled/unskilled), mother’s education, mother’s age, mother’s occupation, father’s education, religion, social class, wealth quintiles and number of living children the women had.

**†**Adjusted for delayed vaccination for DPT–1.

### Additional findings

As part of the exploratory analysis, determinants of full immunization and delayed vaccination were also documented for normal birth weight infants. Lower wealth quintiles, belonging to Muslim community, mother’s age <20 years and female sex were associated with low odds to full immunization, largely similar to that observed in low birth weight infant. Higher maternal education and delivery at a government facility were associated with increased odds of full immunization and decreased odds of delayed vaccination. They are presented in Table S3 and S4 in **Online Supplementary Document(Online Supplementary Document)**.

## DISCUSSION

The present secondary data analysis aimed to understand immunization practices in low birth weight babies and elucidate their determinants. Only a third of LBW infants were fully immunized and majority had delayed vaccination for DPT–1 and DPT–3. The findings pertain to study districts where overall immunization performance is lower compared to other districts of the state. These study districts are recognized as “low performing” by the government of Haryana, based on the indicators for uptake of immunization services [34,35]. However, even though these are “difficult” districts in terms of immunization coverage, this situation is what it would be in many parts of India. The determinants of delay and incomplete immunization that have been identified in this study are over and above the health system’s issues.

The strength of this study is the robust population– based surveillance system and low loss to follow up. All babies were recruited within 72 hours of birth and weight measured by trained study team, thereby reducing chances of misclassification of infants by birth weight. To achieve adequate quality of data on vaccination status, the study team members were rigorously trained and underwent periodic inter and intra observer standardization exercises [23].

A limitation that must be considered while interpreting the findings is that the main trial excluded sick babies or those that were unable to feed. Such babies would include a certain proportion of LBW infants (possibly the smallest/with lowest birth weight) and in them, the delay and incompleteness in vaccination may be possibly of greater magnitude. Excluding them, therefore, may underestimate the actual delay and incompleteness in immunization. Also, in this setting, we recognize that a small proportion of pregnant women, especially those having the first baby, tend to go to their parents home for delivery and these were therefore not available for enrolment. The immunization practices of these primigravida mothers could be different from those who would have had children previously and this might have possibly affected the findings observed. There was no reliable data on gestational age and so through the current analysis, it would be difficult to interpret whether the immunization practices were influenced by prematurity or not. In around one–fifth of the infants, data on immunization was obtained through reliable history instead of documented evidence in form of immunization card. Thus, the possibility of reporting inaccurate vaccination dates cannot be ruled out. Other factors that could affect immunization uptake such as maternal illness and distance from the health facility were not considered as data was unavailable for these variables. Delayed immunization and low rates of full immunization could also be due to factors affecting supply ie, shortage of vaccines and skilled manpower and other logistic issues but these have not been considered in the current analysis.

After adjustment for potential confounders, being born with low birth weight emerged as a significant determinant of full immunization, and in male infants, also for delayed vaccination with DPT–1 and DPT–3. Interestingly, it was not associated with delay for either DPT–1 or DPT–3 vaccination in females. It could possibly mean that family members/caregivers might hesitate vaccinating their LBW infant, early in life, as they are considered fragile and this fear may be more for male babies, as they are valued more in a patriarchal society like that of Haryana. Lower wealth quintiles, Muslim religion and young maternal age (<20 years) were found to be associated with lower odds of full immunization and higher odds of delayed vaccination for DPT–1 in the final multivariable model. This is in concordance with findings from earlier studies [21,36–38]. Belonging to a lower wealth quintile might represent limited financial ability to access quality health care services whereas young maternal age may suggest mother’s lack of knowledge and preparedness towards adequate care of the infant [39]. Previous studies have documented minority religions such as Muslims as a subset of population that are resistant to uptake of immunization services, as they consider it to be detrimental to the infant’s health [36,37].

In the final regression model; female sex of the infant, delivery by an unskilled attendant and increasing number of children a woman had were also associated with low odds of full immunization. Social constructs in traditional Indian society subject females towards unequal treatment, notably in the state of Haryana. Studies have reported a household level gender– based differential in terms of allocation of food, care seeking and education, usually with the female child being neglected [40–42]. Delivery by an unskilled attendant might be considered as initial cue towards inadequate health care seeking behaviour of the family. The opportunity for an initial exposure to desired and recommended child care practices through a skilled birth attendant is usually lost when delivery is conducted by unskilled personnel. With increasing number of siblings, the infant was less likely to be fully immunized. This could be attributed to the possible increase in responsibilities for the mother, leading to limited attention to the infant. Previous studies have cited “mothers being busy” as an important reason for inadequate immunization practices for their children [20,43].

Similar to previous studies, in this study as well, high maternal education was found to be strongly associated with improved vaccination status of the infant [44,45]. Increasing access to education for girls and young women is clearly a priority. It will produce multiple benefits for health and development, as well as support sustained improvement in infant and child care practices. From a short term perspective, even targeted health literacy interventions in mothers, irrespective of their education status, could improve child care practices including appropriate uptake of immunization services. Increased maternal age (mainly 30 years and older) had higher odds of full immunization. This might be due to experience accrued by the mother with time on benefits of immunization. Delivery at a government health facility was associated not only with increased odds of full immunization but also with lower odds of delayed vaccination. This finding is interesting and reassuring at the same time. Availability of vaccines free of cost in a government facility might have led to improved immunization practices.

## CONCLUSIONS

To the best of our knowledge, it is one of the few data presented from LMIC, particularly in India, to understand the immunization practices in LBW infants and their determinants. The findings show that immunization uptake in these infants was inadequate. Strengthening of essential newborn care practices early in life, with a focus on timely initiation of vaccination and ensuring full immunization should form the linchpin of the low birth weight infant care package. In the current study, poor immunization uptake was observed in the economically weaker sections of the society. This calls for due emphasis on ensuring equity in terms of utilization of immunization services and improving coverage.

Data surveillance and monitoring should routinely focus on identifying groups that are underserved by vaccination. Mobilization activities need to focus on infants from the marginalized sections of the society. Interventions aimed at delaying the age at child birth, addressing female bias, providing targeted education on the importance of immunization to mothers of child bearing age and to women of certain religious communities could prove beneficial. Promoting institutional births and emphasizing on immunization as an integral part of the discharge counselling package would be warranted. Interventions that target the determinants should necessarily be accompanied by efforts to improve the health system.
