# Supplementary material for: Immunization practices in low birth weight infants from rural Haryana, India: Findings from secondary data analysis
Source: J Glob Health. 2017 Dec 20;7(2):020415. doi: 10.7189/jogh.07.020415 (PMC5804036; doi:10.7189/jogh.07.020415)
Supplement: Online Supplementary Document [file jogh-07-020415-s001.pdf]

Online Supplementary Document

Upadhyay et al. Immunization practices in low birth weight infants from rural Haryana, India: Findings from secondary data analysis

J Glob Health 2017;7:020415

Supplementary table 1.

Determinants of full immunization at one year of age among low birth weight babies in rural Haryana, North India using data on immunization obtained through vaccination cards

Variables	Unadjusted OR (95% CI)	p-value	Adjusted OR [§] (95% CI)	p-value
**HOUSEHOLD CHARACTERISTICS**				
Quintiles				
1 (Least poor)	Ref		Ref	
2	0.86 (0.73-1.02)	0.079	0.97 (0.82-1.17)	0.813
3	0.61 (0.52-0.73)	<0.001	0.79 (0.65-0.96)*	0.018
4	0.44 (0.36-0.52)	<0.001	0.62 (0.50-0.76)*	<0.001
5 (Poorest)	0.20 (0.16-0.25)	<0.001	0.37 (0.28-0.47)*	<0.001
Religion				
Hindu	Ref		Ref	
Muslim	0.27 (0.22-0.33)	<0.001	0.43 (0.35-0.54)*	<0.001
Others†	0.89 (0.56-1.43)	0.639	1.05 (0.65-1.71)	0.844

Ethnicity				
General	Ref		Ref	
Other Backward Class	0.56 (0.49-0.65)	<0.001	1.07 (0.92-1.24)	0.391
Schedule caste/tribe	0.71 (0.61-0.83)	<0.001	1.11 (0.94-1.31)	0.231
**MATERNAL AND PATERNAL CHARACTERISTICS**				
Mother's age (in years)				
<20	0.67 (0.55-0.83)	<0.001	0.73 (0.59-0.91)*	0.005
20-25	Ref		Ref	
26-30	1.02 (0.87-1.18)	0.837	1.39 (1.17-1.66)*	<0.001
>30	0.57 (0.42-0.78)	<0.001	1.38 (0.96-1.96)	0.076
Mother's education (Years of schooling)				
Illiterate (0)	Ref		Ref	
Less than primary (1 to <5)	1.33 (0.96-1.84)	0.091	1.09 (0.78-1.53)	0.600
Primary completed and secondary incomplete (5 to <12)	2.27 (1.99-2.59)	<0.001	1.34 (1.16-1.56)*	<0.001
Secondary complete and higher education (≥12)	3.91 (3.24-4.71)	<0.001	1.85 (1.47-2.33)*	<0.001
Father's education (years of schooling)				
Illiterate (0)	Ref			
Less than primary (1 to <5)	1.27 (0.89-1.81)	0.180	1.18 (0.82-1.69)	0.369
Primary completed and secondary incomplete (5 to <12)	2.11 (1.71-2.59)	<0.001	1.20 (0.96-1.50)	0.108
Secondary complete and higher education (≥12)	3.24 (2.59-4.06)	<0.001	1.11 (0.85-1.44)	0.449
**BIRTH RELATED CHARACTERISTICS**				
Place of delivery				
Home	Ref		Ref	
Government hospital	1.88 (1.64-2.16)	<0.001	1.23 (1.04-1.54)*	0.038
Private hospital	2.02 (1.75-2.34)	<0.001	1.05 (0.83-1.32)	0.687
Personnel conducting delivery [£]				
Skilled	Ref		Ref	
Unskilled	0.45 (0.39-0.51)	<0.001	0.74 (0.59-0.93)*	0.010
No. of living children				
0	Ref		Ref	
1-2	0.96 (0.84-1.07)	0.449	1.00 (0.87-1.14)	0.995
3-4	0.61 (0.47-0.79)	<0.001	0.76 (0.56-1.02)	0.070
≥4	0.24 (0.16-0.36)	<0.001	0.42 (0.27-0.66)*	<0.001
Singleton	Ref			
Multiple	0.97 (0.72-1.32)	0.867	---	---

**INFANT CHARACTERISTICS**				
Birth weight (in grams)				
2000-2499	Ref		Ref	
<2000	0.95 (0.79-1.16)	0.651	0.96 (0.79-1.17)	0.711
Sex of the baby				
Male	Ref		Ref	
Female	0.87 (0.78-0.98)	0.022	0.87 (0.77-0.97)*	0.023

[§]Variables with p-value <0.20 in the bivariate analysis were included in the multivariable analysis and have been presented in the table, exception being the birth weight which was placed in the multivariable regression model irrespective of the p-values in bivariate analysis as this was considered an essential variable determining delay. Mother's occupation had a p-value of ≥0.20 in bivariate analysis and was not included in the multivariable analysis; †others Christian/Sikh/Jain/Parsi/Zoroastrian/Buddhist/neo Buddhist; £ skilled attendant included doctor/nurse/Auxiliary Nurse Midwife/community health worker; unskilled included traditional birth attendant/relative/neighbour ; * statistically significant at p<0.05

Supplementary table 2.

Determinants of delayed vaccination with first dose DPT at age >10 weeks and third dose DPT at age>18 weeks for low birth weight babies in rural Haryana, North India using data on immunization obtained through vaccination cards

Variables	DPT-1 (at >10 weeks after birth)				DPT-3 (at >18 weeks after birth)			
	Unadjusted OR (95% CI)	p-value	Adjusted OR§ (95% CI)	p-value	Unadjusted OR (95% CI)	p-value	Adjusted OR§ (95% CI)	p-value
**HOUSEHOLD CHARACTERISTICS**								
Quintiles								
1 (Least poor)	Ref		Ref		Ref		Ref	
2	1.26 (1.07-1.48)	0.004	1.14 (0.96-1.35)	0.133	1.19 (0.95-1.51)	0.119	1.06 (0.83-1.35)	0.630
3	1.37 (1.16-1.61)	<0.001	1.19 (0.97-1.40)	0.152	1.11 (0.87-1.41)	0.398	0.91 (0.69-1.19)	0.491
4	1.44 (1.22-1.68)	<0.001	1.26 (1.02-1.51)*	0.045	0.99 (0.78-1.26)	0.938	0.78 (0.58-1.08)	0.105
5 (Poorest)	1.64 (1.36-1.97)	<0.001	1.28 (1.05-1.56)*	0.013	0.98 (0.74-1.31)	0.904	0.72 (0.51-1.05)	0.081
Religion								
Hindu	Ref		Ref		Ref			
Muslim	1.53 (1.27-1.86)	<0.001	1.37 (1.12-1.68)*	0.003	1.14 (0.84-1.56)	0.391	0.94 (0.68-1.31)	0.741
Others†	1.17 (0.78-1.76)	0.458	1.12 (0.74-1.70)	0.594	1.90 (0.91-4.01)	0.090	1.73 (0.82-3.68)	0.150
Ethnicity								
General	Ref		Ref		Ref			
Other Backward Class	1.28 (1.13-1.46)	<0.001	1.09 (0.95-1.25)	0.211	1.24 (1.02-1.49)	0.033	1.16 (0.94-1.42)	0.161
Schedule caste/tribe	1.27 (1.10-1.45)	0.001	1.10 (0.94-1.28)	0.222	0.91 (0.75-1.12)	0.384	0.84 (0.68-1.06)	0.141
**MATERNAL AND PATERNAL CHARACTERISTICS**								
Mother's age (in years)								
<20	1.35 (1.12-1.62)	0.001	1.27 (1.05-1.54)*	0.016	1.24 (0.91-1.68)	0.169	1.16 (0.85-1.59)	0.344
20-25	Ref		Ref		Ref		Ref	
26-30	1.07 (0.93-1.24)	0.343	0.98 (0.84-1.16)	0.876	0.97 (0.78-1.21)	0.824	0.94 (0.74-1.19)	0.608
>30	1.20 (0.91-1.59)	0.197	0.90 (0.64-1.24)	0.511	1.09 (0.69-1.73)	0.684	0.94 (0.57-1.58)	0.840
Mother's education (Years of schooling)								
Illiterate (0)	Ref		Ref		Ref		Ref	
Less than primary (1 to <5)	0.87 (0.65-1.17)	0.354	0.90 (0.67-1.22)	0.497	0.83 (0.52-1.33)	0.435	0.82 (0.51-1.32)	0.416
Primary completed and secondary incomplete (5 to <12)	0.80 (0.71-0.90)	<0.001	0.87 (0.76-0.98)*	0.045	0.94 (0.77-1.14)	0.510	0.87 (0.70-1.08)	0.204

Secondary complete and higher education (≥12)	0.48 (0.40-0.58)	<0.001	0.56 (0.45-0.71)*	<0.001	0.62 (0.49-0.79)	<0.001	0.56 (0.41-0.76)*	<0.001
Father's education (years of schooling)								
Illiterate (0)	Ref		Ref		Ref		Ref	
Less than primary (1 to <5)	1.11 (0.81-1.52)	0.521	1.14 (0.82-1.57)	0.436	0.85 (0.49-1.45)	0.545	0.86 (0.50-1.47)	0.575
Primary completed and secondary incomplete (5 to <12)	0.85 (0.71-1.02)	0.076	1.01(0.83-1.22)	0.948	0.76 (0.55-1.06)	0.104	0.76 (0.54-1.07)	0.116
Secondary complete and higher education (≥12)	0.72 (0.59-0.88)	0.001	1.11 (0.88-1.39)	0.378	0.69 (0.49-0.96)	0.032	0.76 (0.52-1.12)	0.167
**BIRTH RELATED CHARACTERISTICS**								
Place of delivery								
Home	Ref		Ref		Ref		Ref	
Government hospital	0.66 (0.58-0.75)	<0.001	0.71 (0.57-0.86)*	0.001	0.76 (0.62-0.92)	0.005	0.77 (0.56-1.06)	0.115
Private hospital	0.85 (0.74-0.96)	0.013	0.97 (0.79-1.20)	0.835	0.83 (0.67-1.02)	0.071	0.84 (0.61-1.16)	0.294
Personnel conducting delivery Ɨ								
Skilled	Ref		Ref		Ref			
Unskilled	1.34 (1.19-1.51)	<0.001	1.02 (0.83-1.26)	0.828	1.22 (1.01-1.47)	0.041	0.99 (0.71-1.37)	0.946
No. of living children								
0	Ref		Ref		Ref		Ref	
1-2	0.98 (0.88-1.09)	0.731	0.95 (0.84-1.07)	0.399	0.98 (0.83-1.16)	0.860	0.96 (0.80-1.16)	0.701
3-4	1.37 (1.07-1.75)	0.011	1.22 (0.93-1.61)	0.151	1.46 (0.95-2.26)	0.084	1.45 (0.90-2.33)	0.128
≥4	1.45 (1.06-1.99)	0.021	1.25 (0.86-1.81)	0.242	1.01 (0.59-1.73)	0.958	0.97 (0.53-1.79)	0.943
Singleton	Ref				Ref			
Multiple	1.05 (0.82-1.36)	0.694	---	---	1.04 (0.71-1.53)	0.827	---	---
**INFANT CHARACTERISTICS**								
Birth weight (in grams)								
2000-2499	Ref				Ref		Ref	
<2000	1.26 (1.06-1.51)	0.010	1.24 (1.04-1.48)*	0.021	1.10 (0.84-1.45)	0.478	1.07 (0.81-1.42)	0.614
Sex of the baby								
Male	Ref		---	---	Ref		---	---
Female	0.96 (0.86-1.06)	0.398			0.96 (0.81-1.12)	0.593		

§ Variables with p-value <0.20 in the bivariate analysis were included in the multivariable analysis and have been presented in the table, exception being the birth weight which was placed in the multivariable regression model irrespective of the p-values in bivariate analysis as this was considered an essential variable determining delay. Mother's occupation had a p-value of ≥0.20 in bivariate analysis and was not included in the multivariable analysis; Ɨ skilled attendant included doctor/nurse/Auxiliary Nurse Midwife/community health worker; unskilled included traditional birth attendant/relative/neighbour; * statistically significant at p<0.05

Supplementary table 3.

Determinants of full immunization at one year of age among normal birth weight babies in rural Haryana, North India

Variables	Unadjusted OR (95% CI)	p-value	Adjusted OR § (95% CI)	p-value
**HOUSEHOLD CHARACTERISTICS**				
Quintiles				
1 (Least poor)	Ref		Ref	
2	0.66 (0.62-0.71)	<0.001	0.80 (0.75-0.86)*	<0.001
3	0.46 (0.43-0.49)	<0.001	0.65 (0.59-0.70)*	<0.001
4	0.33 (0.31-0.35)	<0.001	0.56 (0.52-0.61)*	<0.001
5 (Poorest)	0.16 (0.15-0.18)	<0.001	0.38 (0.34-0.42)*	<0.001
Religion				
Hindu	Ref		Ref	
Muslim	0.21 (0.19-0.22)	<0.001	0.39 (0.36-0.42)*	<0.001
Others†	0.83 (0.67-1.03)	0.096	1.03 (0.82-1.31)	0.785
Ethnicity				
General	Ref		Ref	
Other Backward Class	0.47 (0.44-0.50)	<0.001	1.06 (0.98-1.13)	0.061
Schedule caste/tribe	0.73 (0.69-0.78)	<0.001	1.25 (1.16-1.34)*	<0.001
**MATERNAL AND PATERNAL CHARACTERISTICS**				
Mother's age (in years)				
<20	0.72 (0.66-0.79)	<0.001	0.68 (0.62-0.76)*	<0.001
20-25	Ref		Ref	
26-30	0.99 (0.94-1.05)	0.916	1.50 (1.40-1.61)*	<0.001
>30	0.66 (0.59-0.73)	<0.001	1.78 (1.57-2.04)*	<0.001
Mother's education (Years of schooling)				
Illiterate (0)	Ref		Ref	
Less than primary (1 to <5)	1.71 (1.51-1.94)	<0.001	1.38 (1.21-1.57)*	<0.001
Primary completed and secondary incomplete (5 to <12)	2.92 (2.76-3.08)	<0.001	1.70 (1.59-1.81)*	<0.001
Secondary complete and higher education (≥12)	5.96 (5.53-6.42)	<0.001	2.49 (2.26-2.74)*	<0.001

Father's education (years of schooling)				
Illiterate (0)	Ref			
Less than primary (1 to <5)	1.16 (1.00-1.35)	0.043	1.05 (0.90-1.23)	0.501
Primary completed and secondary incomplete (5 to <12)	2.42 (2.22-2.63)	<0.001	1.30 (1.19-1.43)*	<0.001
Secondary complete and higher education (≥12)	4.39 (4.02-4.81)	<0.001	1.25 (1.12-1.39)*	<0.001
**BIRTH RELATED CHARACTERISTICS**				
Place of delivery				
Home	Ref		Ref	
Government hospital	1.93 (1.83-2.04)	<0.001	1.17 (1.07-1.28)*	0.001
Private hospital	2.22 (2.10-2.35)	<0.001	0.92 (0.80-1.05)	0.079
Personnel conducting delivery £				
Skilled	Ref		Ref	
Unskilled	0.41 (0.39-0.43)	<0.001	0.70 (0.64-0.77)*	<0.001
No. of living children				
0	Ref		Ref	
1-2	0.72 (0.69-0.76)	<0.001	0.77 (0.73-0.82)*	<0.001
3-4	0.50 (0.46-0.55)	<0.001	0.71 (0.64-0.79)*	<0.001
≥4	0.20 (0.25-0.31)	<0.001	0.56 (0.49-0.65)*	<0.001
Singleton	Ref		Ref	
Multiple	1.84 (1.21-2.79)	0.004	1.48 (0.94-2.33)	0.086
**INFANT CHARACTERISTICS**				
Sex of the baby				
Male	Ref		Ref	
Female	0.87 (0.83-0.91)	<0.001	0.87 (0.82-0.91)*	<0.001

[§]Variables with p-value <0.20 in the bivariate analysis were included in the multivariable analysis and have been presented in the table. Mother's occupation had a p-value of ≥0.20 in bivariate analysis and was not included in the multivariable analysis; †others Christian/Sikh/Jain/Parsi/Zoroastrian/Buddhist/neo Buddhist; £ skilled attendant included doctor/nurse/Auxiliary Nurse Midwife/community health worker; unskilled included traditional birth attendant/relative/neighbour; * statistically significant at p<0.05

Supplementary table 4.

Determinants of delayed vaccination with first dose DPT at age >10 weeks and third dose DPT at age>18 weeks for normal birth weight babies in rural Haryana, North India

**Variables**	**DPT-1 (at >10 weeks after birth)**				**DPT-3 (at >18 weeks after birth)**			
	**Unadjusted OR (95% CI)**	**p-value**	**Adjusted OR§ (95% CI)**	**p-value**	**Unadjusted OR (95% CI)**	**p-value**	**Adjusted OR§ (95% CI)**	**p-value**
**HOUSEHOLD CHARACTERISTICS**								
Quintiles								
1 (Least poor)	Ref		Ref		Ref		Ref	
2	1.35 (1.26-1.45)	<0.001	1.22 (1.13-1.32)*	<0.001	1.42 (1.28-1.56)	<0.001	1.25 (1.12-1.38)*	<0.001
3	1.48 (1.38-1.59)	<0.001	1.28 (1.18-1.39)*	<0.001	1.39 (1.26-1.55)	<0.001	1.16 (1.03-1.31)*	0.012
4	1.66 (1.55-1.79)	<0.001	1.34 (1.22-1.46)*	<0.001	1.45 (1.29-1.63)	<0.001	1.13 (0.97-1.29)	0.077
5 (Poorest)	2.06 (1.89-2.23)	<0.001	1.48 (1.34-1.65)*	<0.001	1.33 (1.16-1.52)	<0.001	0.98 (0.83-1.16)	0.869
Religion								
Hindu	Ref		Ref		Ref			
Muslim	1.92 (1.78-2.06)	<0.001	1.52 (1.39-1.65)*	<0.001	1.56 (1.36-1.80)	<0.001	1.36 (1.16-1.58)*	<0.001
Others†	1.15 (0.92-1.44)	0.226	1.05 (0.83-1.33)	0.655	1.26 (0.87-1.81)	0.206	1.17 (0.81-1.69)	0.394
Ethnicity								
General	Ref		Ref		Ref			
Other Backward Class	1.38 (1.31-1.46)	<0.001	1.01 (0.95-1.08)	0.677	1.36 (1.25-1.48)	<0.001	1.16 (1.06-1.27)*	0.002
Schedule caste/tribe	1.18 (1.11-1.26)	<0.001	0.94 (0.88-1.02)	0.121	1.21 (1.11-1.33)	<0.001	1.06 (0.95-1.17)	0.290
**MATERNAL AND PATERNAL CHARACTERISTICS**								
Mother's age (in years)								
<20	1.44 (1.31-1.59)	<0.001	1.42 (1.28-1.57)*	<0.001	1.39 (1.17-1.66)	<0.001	1.43 (1.19-1.71)*	<0.001
20-25	Ref		Ref		Ref		Ref	
26-30	0.92 (0.86-0.97)	0.005	0.81 (0.76-0.86)*	<0.001	0.85 (0.77-0.93)	<0.001	0.77 (0.69-0.85)*	<0.001
>30	1.02 (0.91-1.14)	0.706	0.75 (0.66-0.85)*	<0.001	0.73 (0.61-0.85)	<0.001	0.59 (0.48-0.71)*	<0.001
Mother's education (Years of schooling)								
Illiterate (0)	Ref		Ref		Ref		Ref	

Less than primary (1 to <5)	0.95 (0.84-1.09)	0.501	1.02 (0.88-1.16)	0.812	1.06 (0.85-1.33)	0.568	1.06 (0.85-1.33)	0.592
Primary completed and secondary incomplete (5 to <12)	0.72 (0.68-0.76)	<0.001	0.85 (0.79-0.91)*	<0.001	0.88 (0.81-0.96)	0.009	0.91 (0.82-1.02)	0.066
Secondary complete and higher education (≥12)	0.45 (0.41-0.48)	<0.001	0.60 (0.55-0.67)*	<0.001	0.56 (0.51-0.63)	<0.001	0.67 (0.58-0.76)*	<0.001
Father's education (years of schooling)								
Illiterate (0)	Ref		Ref		Ref		Ref	
Less than primary (1 to <5)	0.95 (0.82-1.09)	0.484	0.96 (0.83-1.11)	0.578	1.29 (0.98-1.70)	0.063	1.26 (0.95-1.65)	0.101
Primary completed and secondary incomplete (5 to <12)	0.73 (0.67-0.79)	<0.001	0.92(0.84-1.03)	0.093	0.95 (0.82-1.10)	0.496	1.03 (0.88-1.21)	0.677
Secondary complete and higher education (≥12)	0.59 (0.53-0.64)	<0.001	1.02 (0.92-1.14)	0.685	0.78 (0.67-0.90)	0.001	1.11(0.92-1.32)	0.249
**BIRTH RELATED CHARACTERISTICS**								
Place of delivery								
Home	Ref		Ref		Ref		Ref	
Government hospital	0.63 (0.59-0.67)	<0.001	0.80 (0.73-0.88)*	<0.001	0.73 (0.67-0.79)	<0.001	0.80 (0.69-0.93)*	0.003
Private hospital	0.66 (0.63-0.71)	<0.001	0.99 (0.91-1.10)	0.973	0.69 (0.64-0.76)	<0.001	0.86 (0.74-1.04)	0.104
Personnel conducting delivery [Ɨ]								
Skilled	Ref		Ref		Ref			
Unskilled	1.63 (1.55-1.72)	<0.001	1.22 (1.11-1.34)*	<0.001	1.39 (1.27-1.52)	<0.001	1.03 (0.88-1.19)	0.736
No. of living children								
0	Ref		Ref		Ref		Ref	
1-2	1.12 (1.06-1.17)	<0.001	1.08 (0.97-1.21)	0.127	1.17 (1.08-1.26)	<0.001	1.21 (1.11-1.32)*	<0.001
3-4	1.22 (1.11-1.34)	<0.001	1.09 (1.04-1.17)*	0.002	1.24 (1.07-1.44)	0.005	1.30 (1.10-1.55)*	0.002
≥4	1.46 (1.31-1.62)	<0.001	1.15 (1.03-1.32)*	0.037	1.18 (0.98-1.41)	0.072	1.29 (1.04-1.60)*	0.023
Singleton	Ref				Ref			
Multiple	0.86 (0.55-1.35)	0.512	---	---	0.59 (0.33-1.05)	0.073	0.68 (0.38-1.21)	0.187
**INFANT CHARACTERISTICS**								
Sex of the baby								
Male	Ref		Ref		Ref		Ref	
Female	1.15 (1.09-1.20)	<0.001	1.14 (1.08-1.19)*	<0.001	1.12 (1.04-1.20)	0.003	1.12 (1.04-1.21)*	0.003

§ Variables with p-value <0.20 in the bivariate analysis were included in the multivariable analysis and have been presented in the table; Mother's occupation had a p-value of ≥0.20 in bivariate analysis and was not included in the multivariable analysis; Ɨ skilled attendant included doctor/nurse/Auxiliary Nurse Midwife/community health worker; unskilled included traditional birth attendant/relative/neighbour; * statistically significant at p<0.05
